# Supplementary material for: Hierarchy of TGFβ/SMAD, Hippo/YAP/TAZ, and Wnt/β-catenin signaling in melanoma phenotype switching
Source: Life Sci Alliance. 2021 Nov 24;5(2):e202101010. doi: 10.26508/lsa.202101010 (PMC8616544; doi:10.26508/lsa.202101010)
Supplement: Supplementary file 2 [file LSA-2021-01010_TableS2.docx]

Table S2. siRNAs

| Product | Supplier | Catalog number or sequences |
| --- | --- | --- |
| ON-TARGET plus non-targeting pool | Dharmacon | D-001810-10 |
| ON-TARGETplus Human LATS1 siRNA SMARTpool | Dharmacon | L-004632-00 |
| ON-TARGETplus Human LATS2 siRNA SMARTpool | Dharmacon | L-003865-00 |
| siGENOME Human CTNNB1 siRNA SMARTpool | Dharmacon | M-003482-00 |
| ON-TARGETplus, Human SMAD4 siRNA SMARTpool | Dharmacon | L-003902-00 |
| ON-TARGETplus, Human WWTR1 siRNA SMARTpool | Dharmacon | L-016083-00 |
| ON-TARGETplus, Human YAP1 siRNA SMARTpool | Dharmacon | L-012200-00 |
| ON-TARGETplus, Human LEF1 siRNA SMARTpool | Dharmacon | L-015396-00-0005 |
| ON-TARGETplus, Human TCF7L2 siRNA SMARTpool | Dharmacon | L-003816-00-0005 |
| AllStars Negative Control siRNA | Qiagen | 1027280 |
| siRNA targeting human CDH2 | Qiagen | Custom-made:  GUGGAGAACCCCAUUGACA |
| Microsynth Control siRNA | Microsynth | AGGUAGUGUAAUCGCCUUG |
| siRNA targeting human CTNNB1 | Microsynth | Custom-made:  1: UAAGCCGGCUAUUGUAGAA  2: GCCACAAGAUUACAAGAAA  3: UGAGACUGCUGAUCUUGGA |
| siRNA targeting human YAP1 | Microsynth | Custom-made:  1: GUAGUUUAGUGUUCUAGAA  2: UUAUUGCCACAUACUCUAA  3: UGACCAUAUUAGUGAAUCU |
