# Supplementary material for: Hierarchy of TGFβ/SMAD, Hippo/YAP/TAZ, and Wnt/β-catenin signaling in melanoma phenotype switching
Source: Life Sci Alliance. 2021 Nov 24;5(2):e202101010. doi: 10.26508/lsa.202101010 (PMC8616544; doi:10.26508/lsa.202101010)
Supplement: Supplementary file 3 [file LSA-2021-01010_TableS3.docx]

Table S3. Primers for quantitative RT-PCR

| Target | Forward primer | Reverse primer |
| --- | --- | --- |
| **ANKRD1** | CATTTGGCAATTGTGGAGAAG | TGGCTGTGGATTCAAGCATA |
| **AXIN2** | CGGAAACTGTTGACAGTGGA | CACTGGATATCTCACTGTCGTTG |
| **CTGF** | CTCCTGCAGGCTAGAGAAGC | GATGCACTTTTTGCCCTTCTT |
| **CDH2** | TAGTCACCGTGGTCAAACCAAT | GTGCTGAATTCCCTTGGCTAAT |
| **CTLA4** | TTCATCCCTGTCTTCTGC | AGTGGCTTTGCCTGGAGA |
| **CTNNB1** | TGTTAAATTCTTGGCTATTACGAC | CCACCACTAGCCAGTATGATGA |
| **CYR61** | GGTCAAAGTTACCGGGCAGT | GGAGGCATCGAATCCCAGC |
| **FN1** | GAACTATGATGCCGACCAGAA | GGTTGTGCAGATTTCCTCGT |
| **LATS1** | CTCTGCACTGGCTTCAGATG | TCCGCTCTAATGGCTTCAGT |
| **LATS2** | ACATTCACTGGTGGGGACTC | GTGGGAGTAGGTGCCAAAAA |
| **LEF1** | TGCATCAGGTACAGGTCCAAG | TCTCCAGAAGAGGTCCTGGG |
| **MITF** | CATTGTTATGCTGGAAATGCTAGA | TGCTAAAGTGGTAGAAAGGTACTGC |
| **MLANA** | GAGAAAAACTGTGAACCTGTGGT | GACTGTTCTGCAGAGAGTTTCTCAT |
| **NKD1** | TCCAAGAAGCAGCTGAAGTTT | GTCATACAGGGTGAAGGTCCA |
| **NOTUM** | ACAGGGATCCTGTCCTCACA | CTCCAAACATCACTGGAGCA |
| **P21** | TCACTGTCTTGTACCCTTGTGC | GGCGTTTGGAGTGGTAGAAA |
| **RPL19** | GATGCCGGAAAAACACCTTG | TGGCTGTACCCTTCCGCTT |
| **SERPINE1** | CTCCTGGTTCTGCCCAAGT | CAGGTTCTCTAGGGGCTTCC |
| **SMAD4** | CCTGTTCACAATGAGCTTGC | GCAATGGAACACCAATACTCAG |
| **SMAD7** | AGGGGGAACGAATTATCTGG | CACAGTAGAGCCTCCCCACT |
| **SNAI1** | GCTGCAGGACTCTAATCCAGA | ATCTCCGGAGGTGGGATG |
| **SNAI2** | TGGTTGCTTCAAGGACACAT | GTTGCAGTGAGGGCAAGAA |
| **TCF7L2 (TCF4)** | TCCTTGAGGGCTTGTCTACTC | AAACGATTGAACACCAGATTGC |
| **TAZ** | TATCCCAGCCAAATCTCGTG | TTCTGCTGGCTCAGGGTACT |
| **TWIST1** | AAGGCATCACTATGGACTTTCTCT | GCCAGTTTGATCCCAGTATTTT |
| **YAP1** | CCTTCTTCAAGCCGCCGGAG | CAGTGTGCCAGGAGAAACAGC |
| **ZEB1** | GCCAACAGACCAGACAGTGTT | TCTTGCCCTTCCTTTCCTG |
| **ZEB2** | CGATCCAGACCGCAATTAAC | TGCTGACTGCATGACCATC |
