# Supplementary material for: Hierarchy of TGFβ/SMAD, Hippo/YAP/TAZ, and Wnt/β-catenin signaling in melanoma phenotype switching
Source: Life Sci Alliance. 2021 Nov 24;5(2):e202101010. doi: 10.26508/lsa.202101010 (PMC8616544; doi:10.26508/lsa.202101010)
Supplement: Supplementary file 4 [file LSA-2021-01010_TableS4.docx]

Table S4. Primary antibodies for immunoblotting

| Product | Supplier | Catalog number | Dilution |
| --- | --- | --- | --- |
| Rabbit anti-GAPDH | Abcam | ab9485 | 1:2500 |
| Rabbit anti-Lats1 | Cell Signaling | 9153 | 1:1000 |
| Mouse anti-Lats2 | Santa Cruz Biotechnology | SC-515579 | 1:500 |
| Mouse anti-Smad4 | Santa Cruz Biotechnology | SC-7966 | 1:750 |
| Mouse anti-β-catenin clone 14/Beta-Catenin | BD Biosciences | 610154 | 1:2000 |
| Mouse anti-TAZ clone M2-616 | BD Biosciences | 560235 | 1:1000 |
| Rabbit anti-YAP | Cell Signaling | 4912 | 1:1000 |
| Rabbit anti-TCF4/TCF7L2 | Cell Signaling | 2569 | 1:1000 |
| Rabbit anti-LEF1 | Abcam | ab137872 | 1:1000 |
| Mouse anti-N-cadherin clone 32/N-Cadherin | BD Biosciences | 610921 | 1:2500 |
